# Genome-wide association study identifies new loci associated with risk of HBV infection and disease progression

**DOI:** 10.1186/s12920-021-00907-0

**Published:** 2021-03-18

**Authors:** Zheng Zeng, Hankui Liu, Huifang Xu, Haiying Lu, Yanyan Yu, Xiaoyuan Xu, Min Yu, Tao Zhang, Xiulan Tian, Hongli Xi, Liping Guan, Jianguo Zhang, Stephen J. O’Brien, the HBVstudy consortium

**Affiliations:** 1 Department of Infectious Diseases, Peking University First Hospital, Beijing, 100034 China; 2 BGI-Shenzhen, Shenzhen, 518083 China; 3 Laboratory of Genomic Diversity, Center for Computer Technologies, ITMO University, St. Petersburg, Russia 197101; 4 Guy Harvey Oceanographic Center, Halmos College of Natural Sciences and Oceanography, Nova Southeastern University, Ft Lauderdale, FL 33004 USA

**Keywords:** HBV infection, Disease progression, GWAS, Host genetic factors, SNPs

## Abstract

**Background:**

Recent studies have identified susceptibility genes of HBV clearance, chronic hepatitis B, liver cirrhosis, hepatocellular carcinoma, and showed the host genetic factors play an important role in these HBV-related outcomes.

**Methods:**

Collected samples from different outcomes of HBV infection and performed genotyping by Affymetrix 500 k SNP Array. GCTA tool, PLINK, and Bonferroni method were applied for analysis of genotyping and disease progression. ANOVA was used to evaluate the significance of the association between biomarkers and genotypes in healthy controls. PoMo, F_ST,_ Vcftools and Rehh package were used for building the racial tree and population analysis. F_ST_ statistics accesses 0.15 was used as a threshold to detect the signature of selection.

**Results:**

There are 1031 participants passed quality control from 1104 participants, including 275 HBV clearance, 92 asymptomatic persistence infection (ASPI), 93 chronic hepatitis B (CHB), 188 HBV-related decompensated cirrhosis (DC), 214 HBV-related hepatocellular carcinoma (HCC) and 169 healthy controls (HC). In the case–control study, one novel locus significantly associated with CHB (SNP: rs1264473, Gene: *GRHL2*, *P* = 1.57 × 10^−6^) and HCC (SNP: rs2833856, Gene: *EVA1C*, *P* = 1.62 × 10^−6^; SNP: rs4661093, Gene: *ETV3*, *P* = 2.26 × 10^−6^). In the trend study across progressive stages post HBV infection, one novel locus (SNP: rs1537862, Gene: *LACE1*, *P* = 1.85 × 10^−6^), and three MHC loci (*HLA-DRB1, HLA-DPB1, HLA-DPA2*) showed significant increased progressive risk from ASPI to CHB. Underlying the evolutionary study of HBV-related genes in public database, the derived allele of two HBV clearance related loci, rs3077 and rs9277542, are under strong selection in European population.

**Conclusions:**

In this study, we identified several novel candidate genes associated with individual HBV infectious outcomes, progressive stages, and liver enzymes. Two SNPs that show selective significance (*HLA-DPA1*, *HLA-DPB1*) in non-East Asian (European, American, South Asian) versus East Asian, indicating that host genetic factors contribute to the ethnic disparities of susceptibility of HBV infection. Taken together, these findings provided a new insight into the role of host genetic factors in HBV related outcomes and progression.

**Supplementary information:**

The online version contains supplementary material available at 10.1186/s12920-021-00907-0.

## Background

Hepatitis B virus (HBV) infection is one of the most common infectious diseases, with about 248 million HBsAg positive individuals worldwide and the largest HBsAg positive population in China [1]. HBV infection can develop a wide spectrum of liver diseases, including chronic hepatitis B, liver cirrhosis, hepatocellular carcinoma [2–4]. Previous studies showed the host genetic factor played a critical role in HBV infection susceptibility and identified associated SNPs with significant contribution, including major histocompatibility complex (MHC) genes, i.e. *HLA-DPA1* (rs3077), *HLA-DPB1* (rs9277535), *HLA-C* (rs3130542), *HLA-DQ* (rs2856718, rs7453920) [5–7], and non-MHC genes, i.e. *UBE2L3* (rs4821116), *INTS10* (rs7000921) [8, 9]. In advanced stages of HBV disease, host genetic factors influence the outcome of HBV infection [7, 10, 11], including *HLA-DQ* (rs9275319), *HLA-DRB1* (rs2647073, rs3997872), *STAT4* (rs7574865), *C2* (rs9267673), *PNPLA3* (rs738408, rs738409), *SLC17A2* (rs80215559), *HFE* (rs1800562) [12, 13] for liver cirrhosis and *KIF1B* (rs17401966), *HLA-DQA1/DRB1* (rs9272105), *HLA-DQ* (rs9275319), *STAT4* (rs7574865) for hepatocellular carcinoma [14–16]. However, these reported HBV-related genes confer relatively small increments in risk and explain a small proportion of heritability. For example, although MHC genes are important for immune response to HBsAg, more than half the heritability is determined by non-MHC genes [17]. Moreover, previous studies showed that the MHC genes share a common influence on HBV infection, liver cirrhosis, hepatocellular carcinoma [6, 12, 15, 16] as well as associate with different risk in these outcomes [18]; i.e. *HLA-DQ, STAT4, C2, HLA-DRB1* for liver cirrhosis and HCC [12], *HLA-DQ* for CHB [6]. These consistent [12] or different [18] risks indicated shared but also modified effects for progressive HBV-related outcomes. These results raised our interest to identify host genetic factor which increases the risk of progressive stages post HBV infection. To reveal new susceptibility genes for HBV infection and the HBV-related outcomes, we performed a genome-wide association study (GWAS) in 1031 participants, including 275 HBV clearance subjects, 92 asymptomatic persistence infection carriers (ASPI), 93 chronic hepatitis B patients (CHB), 188 HBV-related decompensated cirrhosis patients (DC), 214 HBV-related hepatocellular carcinoma patients (HCC) and 169 healthy controls (HC) (Table 1).

**Table 1:** Characteristics of participants in the genome-wide association cohorts

Disease categories	HBV clearance	ASPI	CHB	DC	HCC	HC
Sample size	275	92	93	188	214	169
Mean age ± SD	49.56 ± 8.8	46.89 ± 6.9 *	46.46 ± 5.7 **	50.65 ± 8.4	51.34 ± 10.5 *	48.82 ± 7.1
Male/female	105/170	33/59	62/31 ***	148/40 ***	183/31 ***	73/96
ALT, U/L, mean ± SD	26.95 ± 30.42	24.5 ± 8.79	169.53 ± 243.76 ***	89.57 ± 115.79 ***	214 ± 381.67 **	23.11 ± 7.97
AST, U/L, mean ± SD	25.89 ± 21.89 *	23.16 ± 6.45	100.04 ± 127.54 ***	102.46 ± 118.36 ***	120.51 ± 286.05 ***	22.23 ± 7.00
TBiL, μmol/L, mean ± SD	13.68 ± 9.93	12.28 ± 2.91	23.12 ± 35.60 ***	65.87 ± 83.22 ***	50.11 ± 90.93 ***	12.95 ± 4.09
DBiL, μmol/L, mean ± SD	5.86 ± 18.39	3.59 ± 1.68	9.36 ± 14.29 **	35.15 ± 51.33 ***	25.87 ± 55.04 ***	4.54 ± 9.46
ALP, U/L, mean ± SD	74.30 ± 35.86	73.57 ± 44.58	108.89 ± 42.20 ***	132.22 ± 61.42 ***	141.63 ± 104.32 ***	70.85 ± 25.33
GGT, U/L, mean ± SD	28.34 ± 30.77	29.88 ± 21.19	88.76 ± 106.63 ***	89.94 ± 132.10 ***	158.08 ± 183.92 ***	27.58 ± 24.88
ALB, g/L, mean ± SD	43.33 ± 6.11	44.16 ± 6.71	42.40 ± 42.25 *	32.28 ± 7.13 **	37.19 ± 7.27 ***	43.93 ± 4.96
AFP, μg/L, mean ± SD	21.39 ± 47.39 *	5.16 ± 4.79	34.00 ± 89.37 *	87.74 ± 169.83 **	7315.22 ± 37,329.94	3.76 ± 4.34
PTA, %, mean ± SD	95.22 ± 52.69	91.67 ± 14.30	90.06 ± 21.76	63.97 ± 28.07 ***	87.31 ± 33.96	90.84 ± 18.71
PLT, 10^9^/L, mean ± SD	147.69 ± 55.40	146.84 ± 44.51	150.27 ± 49.61	73.96 ± 45.30 ***	145.72 ± 79.24	148.94 ± 49.10

Abbreviations: ASPI, asymptomatic persistence infection; CHB, chronic hepatitis B; DC, decompensated cirrhosis; HCC, hepatocellular carcinoma; HC, healthy controls; SD, standard deviation; ALT, alanine aminotransferase; AST, aspartate aminotransferase; TBIL, total bilirubin; DBIL, direct bilirubin; ALP, alkaline phosphatase; GGT, glutamyl transpeptidase

Notes: *, statistical significance of the difference between each case group and HC group (***: p = [0, 0.001], **: p = (0.001, 0.01], *: p = (0.01, 0.05]). The significances of gender and other characteristics were calculated by Fisher's exact test and ANOVA test, respectively

## Methods

### Study participants

A total of 1104 unrelated, age- and gender- matched, Chinese participants were recruited in the study, enrollment criteria were consistent with a previous report [19]. The population of HBV-related phenotypes was composed of five subgroups: HBV clearance subjects, asymptomatic persistence infection (ASPI) carriers, chronic hepatitis B (CHB) patients, HBV-related decompensated cirrhosis (DC) patients, HBV-related hepatocellular carcinoma (HCC) patients. Healthy controls (HC) who were HBV serum marker-negative (HBsAg, anti-HBc) and had no serological evidence of co-infection with HCV, HDV, and HIV were also included. HBV chronic infection patients were diagnosed based on seropositivity of HBsAg at least 6 months. Then ASPI was defined as HBsAg and anti-HBc positive at least 6 months and serum alanine aminotransferase (ALT), aspartate aminotransferase (AST) in normal values without abnormal before. CHB is defined as HBsAg and anti-HBc positive at least 6 months and ALT, AST abnormal before or at enrollment. DC was defined as HBsAg and anti-HBc positive at least 6 months with decompensated portal hypertension (gastroesophageal bleeding, ascites, edema or encephalopathy) or decompensated liver function (albumin < 35 g/L and total bilirubin > 35umol/L). HCC was defined at least one of following: (a) liver biopsy; or (b) abnormal alpha fetoprotein (AFP) and sonographic, CT or MRI space occupying evidence.

### Clinical parameters

Clinical parameters including serum alanine aminotransferase (ALT), aspartate aminotransferase (AST), total bilirubin (TBIL), direct bilirubin (DBIL), alkaline phosphatase (ALP), glutamyl transpeptidase (GGT), albumin (ALB), globulin (Glo), alpha fetoprotein (AFP), prothrombin time activity (PTA), platelets (PLT), HBsAg, anti-HBs, HBeAg, anti-HBe, anti-HBc were collected from hospital information system. Other baseline characteristics were recorded during each patient’s clinical examination. In brief, liver biochemistry and virological tests were carried out by Bechman Coulter AU chemistry analyzers, chemiluminescence immunoassays (AxSYM or ARCHITECT I2000, Abbott, USA) or Ortho/Chemi-luminescent assay (Johnson and Johnson Co., USA) with commercially available kits; Anti-HAV IgM antibody, HDV antigen (HDAg) and anti-HDV antibody, and anti-HEV antibody were determined by commercially ELISA kits in China. For HBV DNA level, it was quantified using commercial real-time polymerase chain reaction kit with a lower limit of detection (LLOD) of 100 IU/ml (Daan company, China) or Roche Cobas Ampliprep/Cobas Taqman™ PCR assay with LLOD of 20 IU/ml (Roche, USA).

### Genome-wide SNP genotyping and quality control

Genotyping was performed on Affymetrix 500k Genome-Wide Human SNP Array 6.0 (http://www.affymetrix.com/Auth/analysis/downloads/na35/genotyping/GenomeWideSNP_6.na35.annot.csv.zip). SNPs met the following quality control procedures were excluded: (1) call rate < 95%; (2) minor allele frequency (MAF) < 1%; (3) genotype in controls deviated from the Hardy Weinberg equilibrium (HWE test P-value < 10^–5^).

### Statistics analysis

GCTA tool [20] was used to perform principal component analyses for estimating population substructure. The first two eigenvectors, pc1 and pc2, were used to display the population structure. PLINK 1.9 [21] software was used to perform logistic regression for identifying susceptibility SNPs of HBV infection and HBV-related outcomes. Gender and age were used as covariates in logistic regression. Chi-square test for trend in proportions was used to identify SNPs with increased effectiveness on disease progression. We used the Bonferroni method to adjust the false positive rate caused by multiple test. The number of independent LD block was used to represent the number of independent multiple test. We calculated a total of 21,077 independent LD blocks via GEC [22] and then set 0.05/21077 as the threshold of genome-wide significance. The genomic control method was used to measure population stratification by calculating the genomic inflation factor (**λ**) from median P-value. ANOVA was used to evaluate the significance of the association between biomarkers and genotypes in healthy controls. Using the SNPs in HBV infection-related loci in 1000 Genomes Project [23], we performed evolutional analyses, including building phylogenetic tree, detecting the signatures of selection, displaying the core haplotypes, estimating effective population size. Derived allele and ancestral allele of SNPs were accessed from Ensemble human ancestral genome (http://ftp.1000genomes.ebi.ac.uk/vol1/ftp/phase1/analysis_results/supporting/ancestral_alignments). PoMo [24], an allele frequency-based approach, was used to build the racial tree based on the allele frequency of SNPs in each population. F_ST_ [25], a classical metrics of population differentiation, was widely employed in detecting signatures of selection [26] in human genome [27, 28] and animal genome [29–31]. In our study, F_ST_ was implemented to detect the selective signature between East Asian population and each other population. Vcftools [32] was used to calculate the F_ST_ statistics of SNPs in paired populations. F_ST_ statistics accesses 0.15 [33] was used as a threshold to detect the signature of selection. Rehh package [34, 35] was used to display the haplotype bifurcation diagrams of the associated SNPs in different populations. Relate [36], a method for genome-wide genealogy estimation for thousands of samples, was used to estimate the historical population size at default setting.

## Results

There are 1031 participants passed quality control from 1104 participants. The demographic and clinical characteristics of 1031 study participants included in our association study are presented in Table 1. All participants were genotyped by Affymetrix 500k SNP Array. A total of 607,153 SNPs passed through quality control (Additional file 1: Figure S1). These SNPs filtered minor allele frequency of < 1% and a call rate of < 95%.

**Fig. 1:**
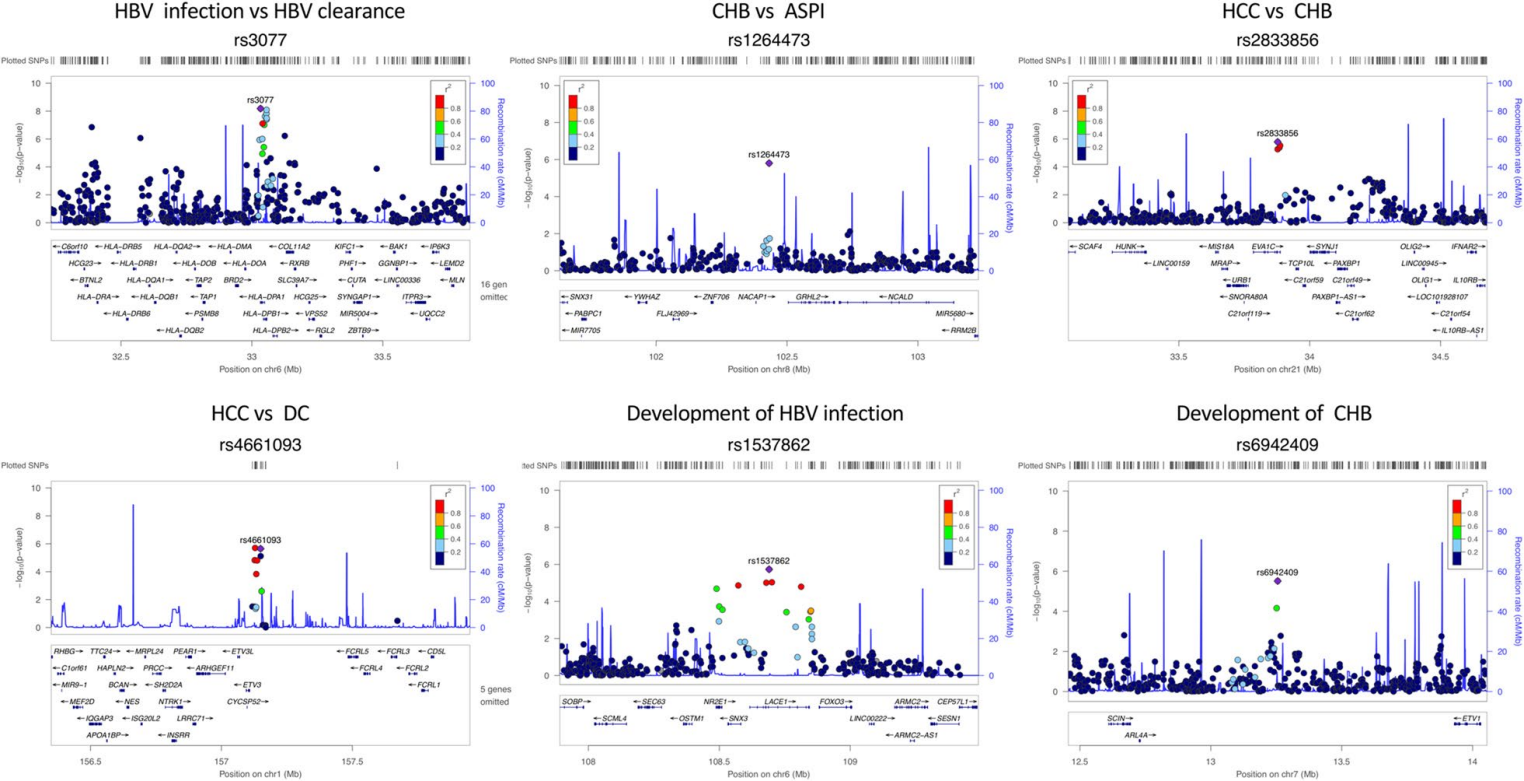
Regional plots shown –log10 *P*-values of SNPs in association study. Marker SNPs are shown as purple diamonds, other SNPs are shown as dots. R-square of Marker SNPs and other SNPs are shown against dark blue, blue, green, yellow and red colors, indicating the linkage disequilibrium. The structure of genes within the region are shown as rectangles and arrows. Abbreviation: ASPI, asymptomatic persistence infection; CHB, chronic hepatitis B; DC, decompensated cirrhosis; HCC, hepatocellular carcinoma

**Fig. 2:**
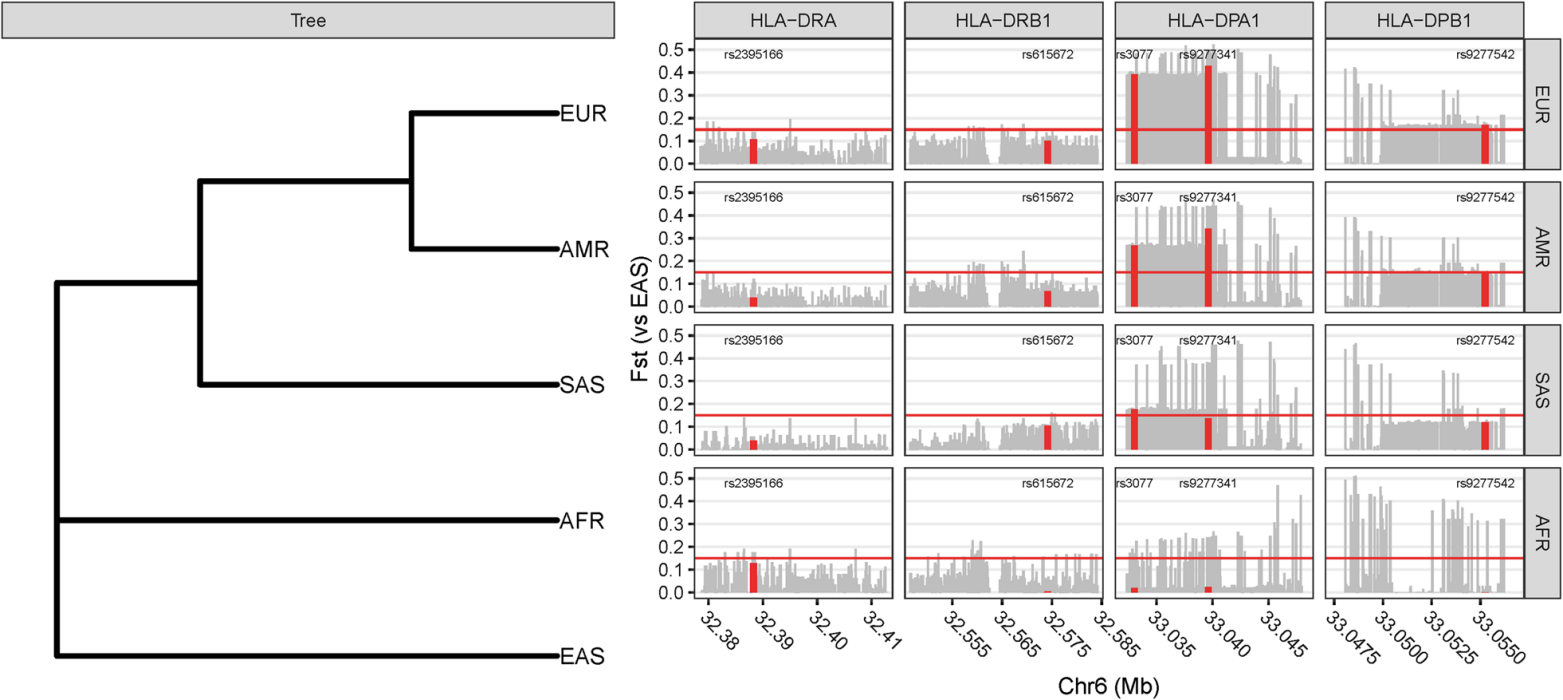
The racial tree (Left) was based on the SNPs in HBV-infection related genes, including *HLA-DRA, HLA-DRB1, HLA-DPA1* and *HLA-DPB1*. The genotype and minor allele frequency of each SNPs were accessed from 1000 Genome Project. EAS, AFR, SAS, EUR, AMR refer to East Asian, African, South Asian, European and American of 1000 Genome Project, respectively. The racial tree indicated a genetic difference in HBV-infection related genes among EAS, AFR, SAS, EUR, AMR. The genetic difference (Right) of each SNPs was evaluated by F_ST_ value. X-axis refer to physical position in chromosome 6. Y-axis refer to F_ST_ value of paired SNP. F_ST_ values of all paired SNPs of AFR, SAS, AMR, EUR versus EAS were displayed in grey bar. F_ST_ values accessed 0.15 (Red horizontal line) indicated the signal of selective event. Red bars and rs IDs showed the reported HBV infection-related SNPs. The F_ST_ values of European versus East Asian showed the genetic difference in *HLA-DPA1* and *HLA-DPB1*, indicating a genetic selection against the HBV infection. The racial tree showed that the East Asian population is at the root, indicating that why we used East Asian population as a comparative population but not the other population, and compared other four populations with East Asian population

**Fig. 3:**
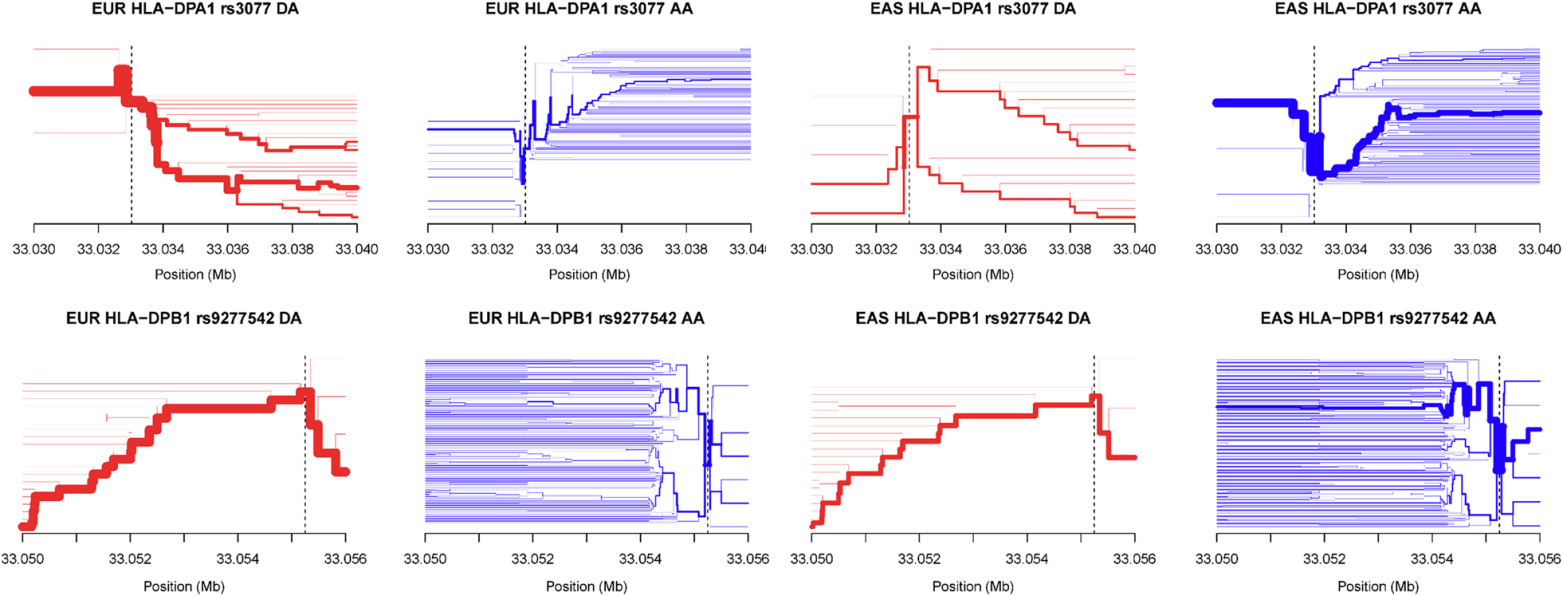
Haplotype bifurcation diagrams of infection-related SNPs, including rs3077 on *HLA-DPA1* (upper) and rs9277542 on *HLA-DPB1* (lower), in European (left) and East Asian (right). EUR and EAS in plot title refer to European and East Asian. DA and AA in plot title refer to derived allele (red) and ancestral allele (blue). Black dash line refers to the position of core SNP. Each node refers to a haplotype. The edge width reflects the population-specific frequency. Haplotype bifurcation diagrams were displayed via Rehh package. Haplotype bifurcation diagrams showed that the derived allele led to a long-range high frequency haplotype in European population and the ancestral allele led to a high frequency haplotype in East Asian population; the ancestral allele led to more haplotypes than the derived allele. The long-range high frequency haplotype confirms the genetic selection in *HLA-DPA1* and *HLA-DPB1* in European population

To demonstrate that there is no genetic stratification in the population, we performed a principal component analysis on the SNPs of all participants. The first two principal components show absence of population structure (Additional file 1: Figure S2). To identify susceptibility SNPs for HBV infection, we performed a GWAS in HBV infection similar with previous design [8, 9]. HBV clearance was used as a control group versus ASPI, CHB, DC, HCC as HBV chronic infection (case group). We observed associations of two novel MHC loci with progression to certain HBV stages (SNP: rs2395166, Gene: *HLA-DRA*, *P* = 1.42 × 10^–7^; SNP: rs615672, Gene: *HLA-DRB1*, *P* = 8.54 × 10^–7^) and two reported MHC loci (SNP: rs3077, Gene: *HLA-DPA1*, *P* = 6.60 × 10^–9^; SNP: rs9277542, Gene: *HLA-DPB1*, *P* = 1.53 × 10^–8^) (Table 2; Fig. 1). These MHC loci variants replicated association results of previous studies affirming that MHC gene alleles confer risks of susceptibility of HBV infection in East Asian. Interestingly, we found that these reported MHC loci (rs2395166:C, rs615672:G, rs3077:A, rs9277542:T, rs9277341:T) present significant differences in allele frequency between East Asian and non-East Asian population in gnomAD database (Table 3), as well as the differences between HBV infection group and HBV clearance group. Since different groups may not present an identical minor allele, here, we used the derived allele against the ancestral allele for studying the allele frequency across different populations. The derived allele frequencies in East Asian are much closer to the HBV chronic infection group, while other populations, such as European, are much closer to the HBV clearance group. These genetic differences may suggest a selective signal in non-East Asian population versus East Asian population. To confirm this, we firstly build a phylogenetic tree based on these loci and then showed the genetic diversity in world-wide populations, in which the East Asian population is at the root. We set the East Asian as the ancestral group in these loci according to the derived allele frequencies and the phylogenetic tree. Subsequently, we identified two strong phylogenetic signals (*HLA-DPA1, HLA-DPB1*) in the European population (Fig. 2) via F_ST_ method. Haplotype bifurcation diagrams of the two core SNPs (rs3077, rs9277542) presented that the resisted allele led to a long-range, and a high frequency homozygosity in European population (Fig. 3), confirming the natural genetic selection. These evidences revealed that the resisted alleles were under positive selection in European population strongly. We estimated the historic population size and then showed these two loci (*HLA-DPA1*, *HLA-DPB1*) were under selection during the past 26,000 years (Additional file 1: Figure S3). These results may provide a context for the racking influence of HBV infectious diseases in history.

**Table 2:** The significance of HBV-related outcomes study

Case–control studies			SNP	Gene	*P* value	OR	Minor Allele	Minor Allele Frequency		Report	λ
Case (n)	Control (n)							Case	Control		
Infection (587)	Clearance (275)		rs2395166	*HLA-DRA*	1.42 × 10^–7^	0.4534	C	0.1269	0.2182	MHC region	1.003
			rs615672	*HLA-DRB1*	8.54 × 10^–7^	0.5697	G	0.405	0.5276	MHC region	
			rs3077	*HLA-DPA1*	6.60 × 10^–9^	0.5007	A	0.2675	0.4145	(Kamatani et al., 2009)	
			rs9277542	*HLA-DPB1*	1.53 × 10^–8^	0.5353	T	0.3735	0.5347	(Kamatani et al., 2009)	
CHB (93)		ASPI (92)	rs1264473	*GRHL2*	1.57 × 10^–6^	3.931	C	0.4402	0.1957	Novel	1.052
HCC (214)	CHB (93)		rs2833856	*EVA1C*	1.62 × 10^–6^	0.3515	C	0.2243	0.4086	Novel	1.022
HCC (214)	DC (188)		rs4661093	*ETV3*	2.26 × 10^–6^	2.841	A	0.2104	0.0882	Novel	1.022

Abbreviations: OR, odds ratio; ASPI, asymptomatic persistence infection; CHB, chronic hepatitis B; DC, decompensated cirrhosis; HCC, hepatocellular carcinoma; MHC, major histocompatibility complex; **λ:** statistics of genomic control

**Table 3:** Divided allele frequency of significant SNPs in MHC region

SNP	Study	Population	Derived Allele	Derived Allele Frequency					*P* value
				Case–control studies			gnomAD		East Asian vs non-East Asian
				Infection	Clearance	Healthy	East Asian	non-East Asian	
rs2395166	our study	Chinese	C	0.127	0.218	0.183	0.128	0.367	4.367 × 10^–195^
rs615672	our study	Chinese	G	0.405	0.528	0.512	0.388	0.590	6.003 × 10^–111^
rs3077	our study	Chinese	A	0.268	0.415	0.379	0.283	0.723	
	Kamatani et al., 2009 [5]	Japanese		0.245	-	0.392			
	Guo et al., 2011 [55]	Chinese		0.314	0.447	0.443			0
	Nishida et al., 2012 [56]	Japanese, Korean		0.213	0.393	-			
	Wong et al., 2013 [57]	Southern Chinese		0.206	0.276	0.288			
rs9277341	our study	Chinese	T	0.142	0.242	0.196	0.159	0.582	0
	Guo et al., 2011 [55]	Chinese		0.133	0.237	0.237			
rs9277542	our study	Chinese	T	0.374	0.535	0.482	0.339	0.627	2.005 × 10^–225^
	Kamatani et al., 2009 [5]	Japanese		0.246	-	0.437			

Abbreviations: East Asian, East Asian population in gnomAD database; non-East Asian, combined all other population excepted East Asian in gnomAD database; *P* value, compared allele frequency between East Asian and non-East Asian population via fisher exact test. Derived Allele was accessed from human ancestral genome (Ensembl-59)

To identify new susceptibility locus for HBV-related outcomes, we performed association studies for CHB, DC, and HCC. Significantly, we observed three associated gene SNP loci: (1) (SNP: rs1264473, Gene: *GRHL2*, *P* = 1.57 × 10^–6^) associated with CHB versus ASPI; (2) (SNP: rs2833856, Gene: *EVA1C*, *P* = 1.62 × 10^–6^) associated with HCC versus CHB; and (3) (SNP: rs4661093, Gene: *ETV3*, *P* = 2.26 × 10^–6^) associated with HCC versus DC (Table 2; Fig. 1). No SNP associated with DC versus CHB were apparent.

**Fig. 4:**
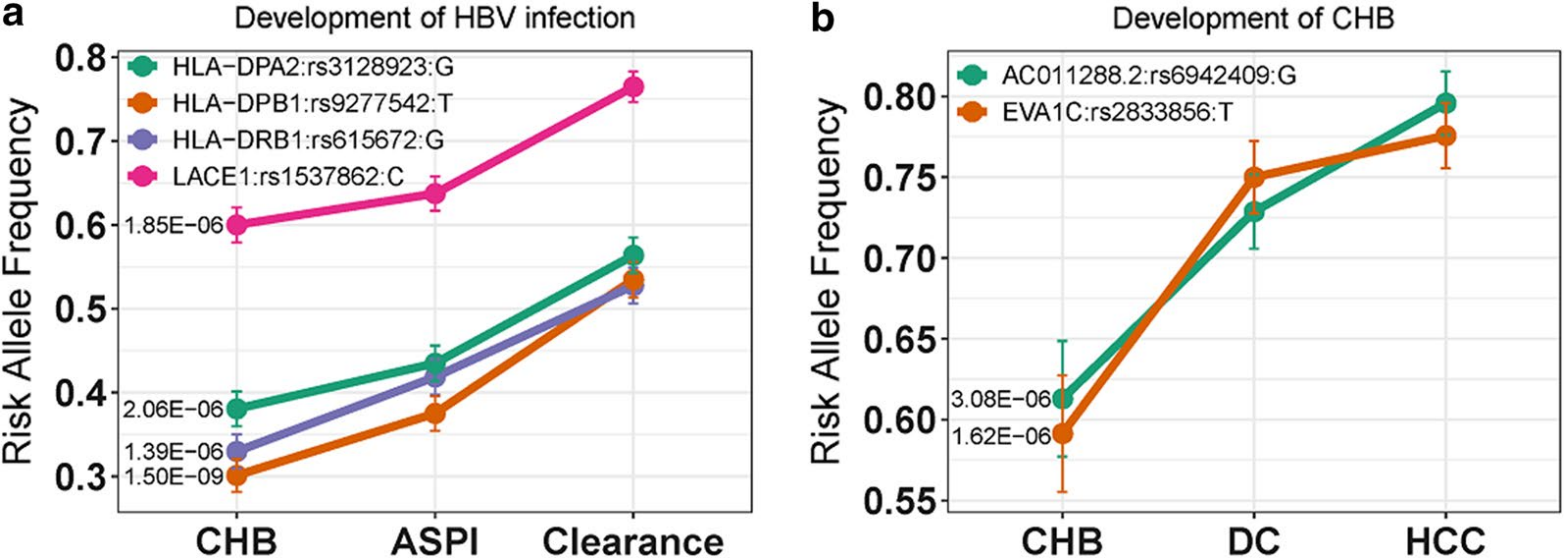
The raising allele frequency in HBV related outcomes during the progression. Four SNPs with increased resistance in CHB, ASPI, HBV clearance during HBV infection (**a**) and two SNPs with increased risk in the CHB, DC, HCC during the development of CHB (**b**). Abbreviation: ASPI, asymptomatic persistence infection; CHB, chronic hepatitis B; DC, decompensated cirrhosis; HCC, hepatocellular carcinoma

HBV clearance, ASPI, CHB, DC, and HCC are progressive stages post HBV infection [4]. We hypothesized that the host genetic factor contributes to the development of outcomes, as well as to the individual outcome. To investigate this hypothesis, we test two progressive stages upon HBV infection: 1.) HBV infection itself (CHB, ASPI, and HBV clearance) and 2.) development of CHB (CHB, DC, and HCC). We performed a chi-square test for trend in proportions of allele to identify SNPs increasing risk of HBV-related outcomes in the progressive stages. We observed association with one novel locus (SNP: rs1537862, Gene: *LACE1*, *P* = 1.85 × 10^–6^), one association with a reported locus (SNP: rs9277542, Gene: *HLA-DPB1*, *P* = 1.50 × 10^–9^), and two association variants at MHC genes (SNP: rs615672, Gene: *HLA-DRB1*, *P* = 1.39 × 10^–6^; SNP: rs3128923, Gene: *HLA-DPA2*, *P* = 2.06 × 10^–6^) with trend test of allele frequency across three outcomes (Table 4; Fig. 4A). The three reported MHC genes were demonstrated to play a critical role in the resistance of HBV infection, and two (*HLA-DPB*:rs9277542, *HLA-DRB1*:rs9277542) were identified to be associated with HBV clearance (Table 2). We did not observe any SNPs achieve genome-wide significant association with development of CHB; One additional locus (SNP: rs6942409, Gene: *AC011288.2*, *P* = 3.08 × 10^–6^) and the HCC associated locus (SNP: rs2833856, Gene: *EVA1C*, *P* = 1.62 × 10^–5^) were associated with increased risk of DC and HCC during the development of CHB (Table 5; Fig. 4b).

**Table 4:** The significance of progressive HBV infection study

SNP	Gene	*P* value	Resistant Allele	Resistant Allele Frequency			Related Risk	
				CHB	ASPI	Clearance	CHB (vs ASPI)	Clearance (vs ASPI)
rs615672	*HLA-DRB1*	1.39 × 10^–6^	G	0.3297	0.4185	0.5276	0.82	1.12
rs9277542	*HLA-DPB1*	1.50 × 10^–9^	T	0.3011	0.375	0.5347	0.84	1.17
rs3128923	*HLA-DPA2*	2.06 × 10^–6^	G	0.3804	0.4348	0.5636	0.89	1.14
rs1537862	*LACE1*	1.85 × 10^–6^	C	0.6	0.6374	0.7647	0.92	1.19

Abbreviations: ASPI, asymptomatic persistence infection; CHB, chronic hepatitis B. RR was calculated with the comparison of CHB and ASPI, Clearance and ASPI respectively

**Table 5:** The suggestive significance of progressive CHB study

SNP	Gene	P value	Risk Allele	Risk Allele Frequency			Related Risk	
				CHB	DC	HCC	DC (vs CHB)	HCC (vs CHB)
rs6942409	*AC011288.2*	3.08 × 10^–6^	G	0.6129	0.7287	0.7958	1.20	1.37
rs2833856	*EVA1C*	1.62 × 10^–5^	T	0.5914	0.75	0.7757	1.30	1.35

Abbreviations: CHB, chronic hepatitis B; DC, decompensated cirrhosis; HCC, hepatocellular carcinoma. RR was calculated with the comparison of HCC and CHB, DC and CHB respectively

**Fig. 5:**
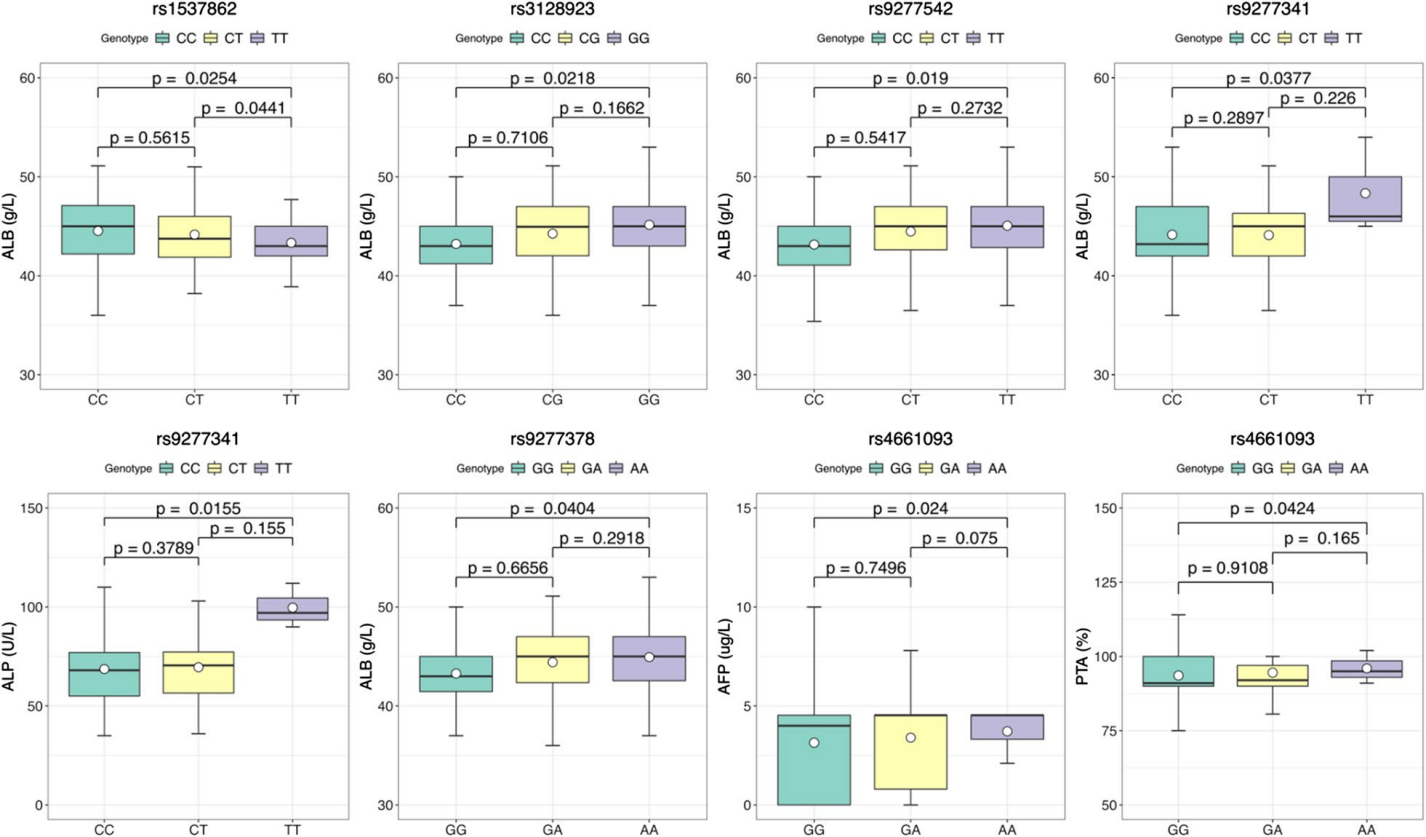
The association between HBV related loci and serum liver enzyme levels in health controls. *P* values were calculated by ANOVA test. White-circle refer to the mean liver enzymes level with different genotypes. The significant differences indicate that these SNPs contribute to liver enzyme activity

Host genetic factors were demonstrated to influence concentrations of liver enzymes in plasma, which are widely used to indicate liver disease [37, 38]. Here, to investigate the functional change in liver influenced by the HBV related loci described above, we performed a variance analysis in 10 clinical parameters of serum liver enzymes (ALT, AST, TBIL, DBIL, ALP, GGT, ALB, AFP, PTA, and PLT) between different genotypes in healthy controls (Additional file 1: Figure S4-9). Six loci (rs1537862, rs3128923, rs9277542, rs9277341, rs9277378, rs4661093) showed modest associations with concentrations of liver enzymes, including ALB, ALP, AFP, and PTA (Fig. 5). These associations suggest pathways linking the host genetic factors, metabolism, and liver function for understanding the mechanisms of infection and disease progression.

In sum, our study identified susceptibility SNPs associated with HBV related outcomes and SNPs increased the risk of progressive outcomes from HBV clearance to HBV chronic infection, DC, and HCC in a Chinese population (Additional file 1: Figure S10).

## Discussion

HBV infection leads to a wide spectrum of clinical outcomes, including spontaneous clearance, asymptomatic carrier, chronic hepatitis B, liver cirrhosis, and hepatocellular carcinoma. Previous studies showed that MHC genes played an important role in outcomes of HBV infection [7]. Alleles associated with HBV infection versus HBV clearance affect infection risk, and a low-risk allele indicated an effect on virus clearance. By contrast loci associated with CHB versus ASPI indicated risk for the severe progression, while a low-risk allele affected tolerance of virus. The tolerance-related gene, *GRHL2*, was demonstrated to influence the inflammation in hepatocytes by regulating microRNA 122 (*MIR122*) and the target of *MIR122*, HIF1α [39]. Levels of *GRHL2* were increased in liver tissues of patients with alcoholic liver disease and correlated with decreases in levels of *MIR122*. Increased levels of *MIR122* in hepatocytes of mice with ethanol-induced liver disease and advanced fibrosis reduced levels of HIF1α and reduced serum levels of alanine aminotransferase (ALT). Taken together, we propose that the low-risk allele rs1264473:T at *GRHL2* ablates severe persistent inflammation through increased the levels of *MIR122*.

Our previous studies [40, 41] showed that *NTCP* S267F mutation significantly affected the disease progression to cirrhosis (*P* = 0.017), and hepatocellular carcinoma (*P* = 0.023) versus CHB [40] and the rs3077:T allele was associated with decreased risk of chronic HBV infection (OR = 0.62, *P* = 0.001) [41]. In this study, we searched for host genetic factor with increased risk of the development-related outcomes in GWAS. One novel locus, *LACE1*, and three infection-related MHC loci were associated the progression of HBV infection. These results showed that the host genetic factors, both MHC and non-MHC genes, increased the risk of progressive outcomes post HBV infection, as well as HBV mutation. It is reported that HBV infection altered the mitochondrial metabolism and mitochondrial dynamics, which result in mitochondrial injury and liver disease [42]. *LACE1* was reported to affect mitochondrial protein homeostasis [43]. Knockdown of *LACE1* converted the expression of a crucial component of regulating mitochondrial dynamics, *OPA1* [43–45]. In addition, we found that the risk allele, *LACE1*:rs1537862:T, decreased the level of ALB significantly (*P* = 0.025, Fig. 5). ALB is a critical marker decreasing with the deterioration of chronic liver diseases [46–48]. Biosynthesis of ALB was affected by proinflammatory cytokines [49, 50] and excess amounts of oxidative agents released by mitochondria from injured liver [46, 51]. Taken together, we proposed *LACE1* may affect hepatic infection by changing the hepatic mitochondrial metabolism and leading to the progression of HBV infection.

There is a limitation in our study, that is we do not have an additional cohort for replicate study. In spite of that, we showed the reported loci in MHC region are significantly related to HBV infection. These replicate results of previous studies confirm our findings are reliable and provide confidence for our study in this cohort. Here, we provide novel candidate genes related to individual outcomes, progressive stages, and liver enzymes. Moreover, we identified two SNPs that show selective significance (*HLA-DPA1*, *HLA-DPB1*) in non-East Asian (European, American, South Asian) versus East Asian. East Asian population seem more susceptible to HBV infection than non-East Asian, and the differences of susceptibility were affected by HBV genotype [52], immunity [53], and environmental exposure [53, 54]. Even in an identical environment (United States), Asian are more prevalent in chronic HBV infection than non-Asian [53]. It seems likely that host genetic factors contribute to the ethnic disparities of susceptibility of HBV infection. Taken together with the genetic associations and evolutionary signals, our findings provide a new insight for HBV study.

## Conclusion

In case–control study, we identified one novel locus (SNP: rs1264473, Gene: *GRHL2*, *P* = 1.57 × 10^–6^) significantly associated with CHB, two novel loci (SNP: rs2833856, Gene: *EVA1C*, *P* = 1.62 × 10^–6^; SNP: rs4661093, Gene: *ETV3*, *P* = 2.26 × 10^–6^) significantly associated with HCC. In trend study across multiple outcomes, we identified one novel locus (SNP: rs1537862, Gene: *LACE1*, *P* = 1.85 × 10^–6^) and three MHC loci (*HLA-DRB1, HLA-DPB1, HLA-DPA2*) significantly increased progressive risk from CHB through ASPI to HBV clearance. In evolutionary study, we showed the derived allele of two HBV clearance related loci, rs3077 and rs9277542, are under strong selection in European population. We suggested these selected alleles may play a role in resisting the susceptibility of HBV in Europeans. Our findings provided a new insight into the role of host genetic factors in HBV related outcomes and progression.

## Supplementary Information

**Additional file 1: Figure S1**. The summary of final SNPs characteristic, including MAF, call rate, and p-value of Hardy-Weinberg equilibrium test.. **Figure S2**. Principal component analyses indicated there are no population stratification among 6 subgroups. Abbreviation: ASPI, asymptomatic persistence infection; CHB, chronic hepatitis B; DC, decompensated cirrhosis; HC: healthy controls; HCC, hepatocellular carcinoma. **Figure S3**. Effective population sizes inferred using Related-package across all individuals of each population in two loci (HLA-DPA1, HLA-DPB1). Recentsize histories (26000 years ago) in European (purple) population showed modest difference compared with East Asian (red) population. Abbreviation: EUR, European; AMR, American; SAS, South Asian; AFR, African.** Figure S4**. Boxplots of rs2395166 genotype and serum liver enzyme levels in HC.** Figure S5**. Boxplots of rs615672 genotype and serum liver enzyme levels in HC. **Figure S6**. Boxplots of rs3077 genotype and serum liver enzyme levels in HC. **Figure S7**. Boxplots of rs1264473 genotype and serum liver enzyme levels in HC. **Figure S8**. Boxplots of rs2833856 genotype and serum liver enzyme levels in HC. **Figure S9**. Boxplots of rs6942409 genotype and serum liver enzyme levels in HC. **Figure S10**. The summary of associated SNPs contributed to HBV-related outcomes and the progression. Abbreviation: PI, persistence infection; ASPI, asymptomatic persistence infection; CHB, chronic hepatitis B; DC, decompensated cirrhosis; HCC, hepatocellular carcinoma.

## Data Availability

The datasets generated and/or analyzed during the current study are not publicly available as they are still being investigated but are available from the corresponding author (Dr. Zheng Zeng) on reasonable request and will be released in China National GeneBank (https://db.cngb.org/cmdb) (need to be approved by Human Genetic Resource Administration of China). The direct web links to the SNPs of 1000 Genomes Project (http://ftp.1000genomes.ebi.ac.uk/vol1/ftp/release/20130502) and Ensemble human ancestral genome (http://ftp.1000genomes.ebi.ac.uk/vol1/ftp/phase1/analysis_results/supporting/ancestral_alignments).

## Abbreviations

AFP: Alpha Fetoprotein
AFR: African
ALB: Albumin
ALP: Alkaline Phosphatase
ALT: Alanine Aminotransferase
AMR: American
anti-HBc: Antibody to Hepatitis B core Antigen
anti-HBe: Antibody to Hepatitis B e Antigen
anti-HBs: Antibody to Hepatitis B surface Antigen
anti-HDV: Antibody to Hepatitis D Virus
anti-HEV: Antibody to Hepatitis E Virus
ASPI: Asymptomatic Persistence Infection
AST: Aspartate Aminotransferase
CHB: Chronic Hepatitis B
DBIL: Direct Bilirubin
DC: Decompensated Cirrhosis
EAS: East Asian
EUR: European
Glo: Globulin
GGT: Glutamyl Transpeptidase
GWAS: Genome Wide Association Study
HBeAg: Hepatitis B e Antigen
HBsAg: Hepatitis B surface Antigen
HBV: Hepatitis B Virus
HCC: Hepatocellular Carcinoma
HC: Healthy Controls
HCV: Hepatitis C Virus
HDV: Hepatitis D Virus
HIV: Human Immunodeficiency Virus
HWE: Hardy–Weinberg Equilibrium
MAF: Minor Allele Frequency
MHC: Major Histocompatibility Complex
PI: Persistence Infection
PLT: Platelets
PTA: Prothrombin Time Activity
SAS: South Asian
SD: Standard Deviation
SNP: Single Nucleotide Polymorphism
TBIL: Total Bilirubin
