# Supplementary material for: Genome-wide association study identifies new loci associated with risk of HBV infection and disease progression
Source: BMC Med Genomics. 2021 Mar 18;14:84. doi: 10.1186/s12920-021-00907-0 (PMC7977299; doi:10.1186/s12920-021-00907-0)
Supplement: Supplementary file 1 — Additional file 1: Figure S1. The summary of final SNPs characteristic, including MAF, call rate, and p-value of Hardy-Weinberg equilibrium test.. Figure S2. Principal component analyses indicated there are no population stratification among 6 subgroups. Abbreviation: ASPI, asymptomatic persistence infection; CHB, chronic hepatitis B; DC, decompensated cirrhosis; HC: healthy controls; HCC, hepatocellular carcinoma. Figure S3. Effective population sizes inferred using Related-package across all individuals of each population in two loci (HLA-DPA1, HLA-DPB1). Recentsize histories (26000 years ago) in European (purple) population showed modest difference compared with East Asian (red) population. Abbreviation: EUR, European; AMR, American; SAS, South Asian; AFR, African. Figure S4. Boxplots of rs2395166 genotype and serum liver enzyme levels in HC. Figure S5. Boxplots of rs615672 genotype and serum liver enzyme levels in HC. Figure S6. Boxplots of rs3077 genotype and serum liver enzyme levels in HC. Figure S7. Boxplots of rs1264473 genotype and serum liver enzyme levels in HC. Figure S8. Boxplots of rs2833856 genotype and serum liver enzyme levels in HC. Figure S9. Boxplots of rs6942409 genotype and serum liver enzyme levels in HC. Figure S10. The summary of associated SNPs contributed to HBV-related outcomes and the progression. Abbreviation: PI, persistence infection; ASPI, asymptomatic persistence infection; CHB, chronic hepatitis B; DC, decompensated cirrhosis; HCC, hepatocellular carcinoma. [file 12920_2021_907_MOESM1_ESM.docx]

**Supplementary Figures**


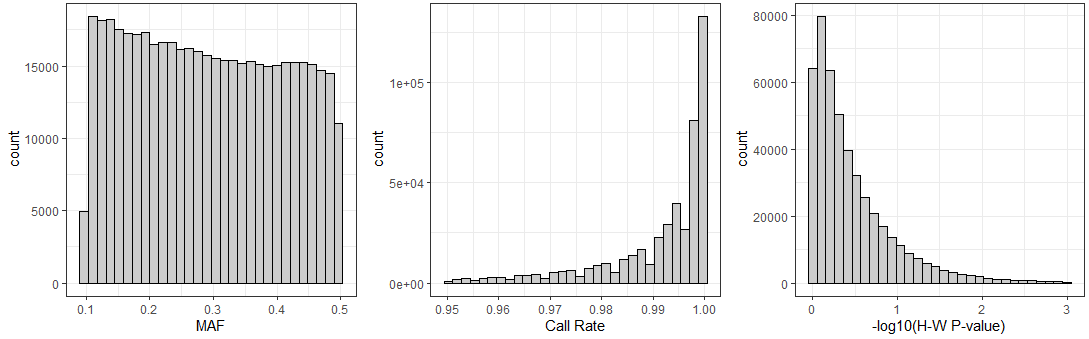


Figure S1: The summary of final SNPs characteristic, including MAF, call rate, and p-value of Hardy-Weinberg equilibrium test.


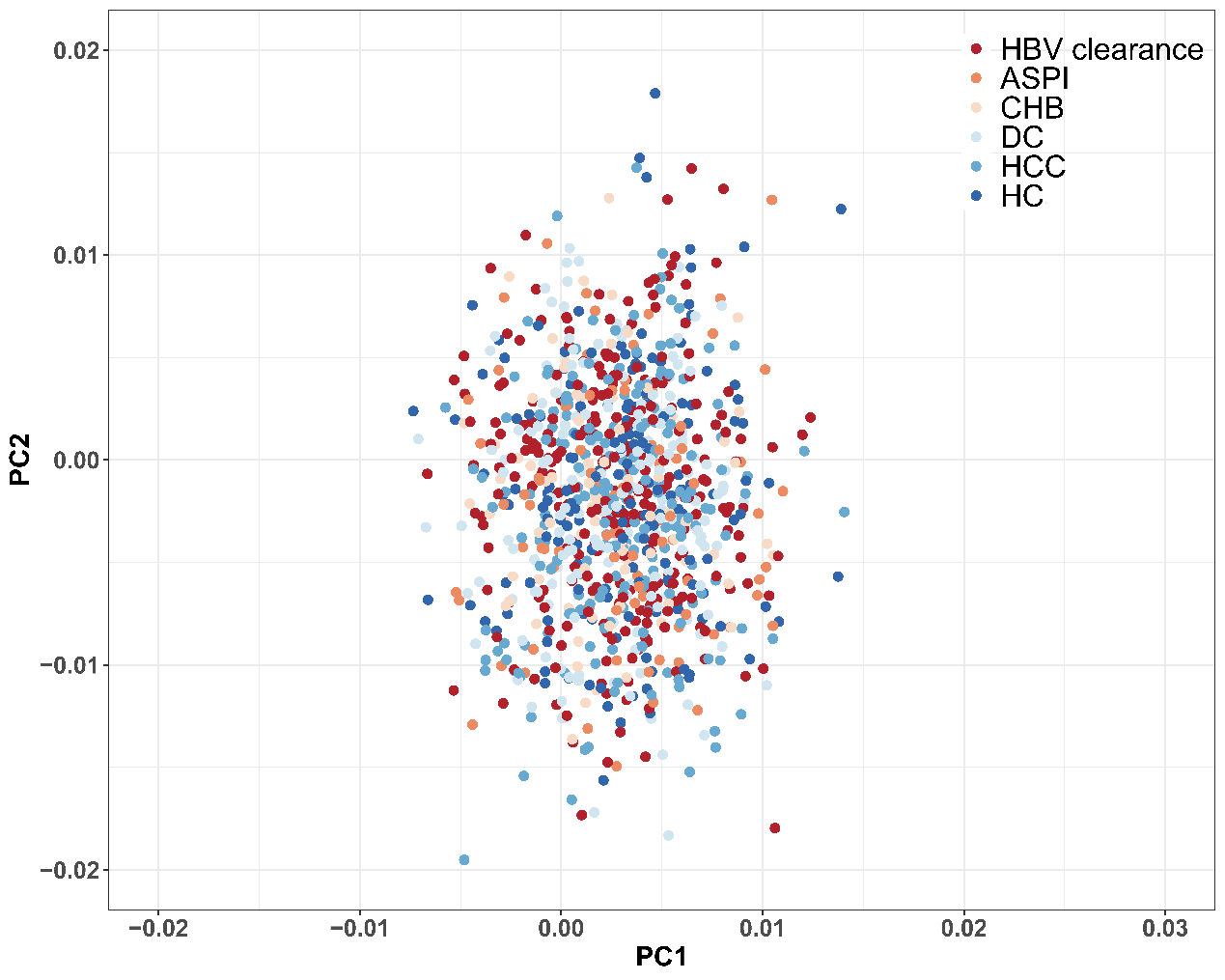


Figure S2: Principal component analyses indicated there are no population stratification among 6 subgroups. Abbreviation: ASPI, asymptomatic persistence infection; CHB, chronic hepatitis B; DC, decompensated cirrhosis; HC: healthy controls; HCC, hepatocellular carcinoma.


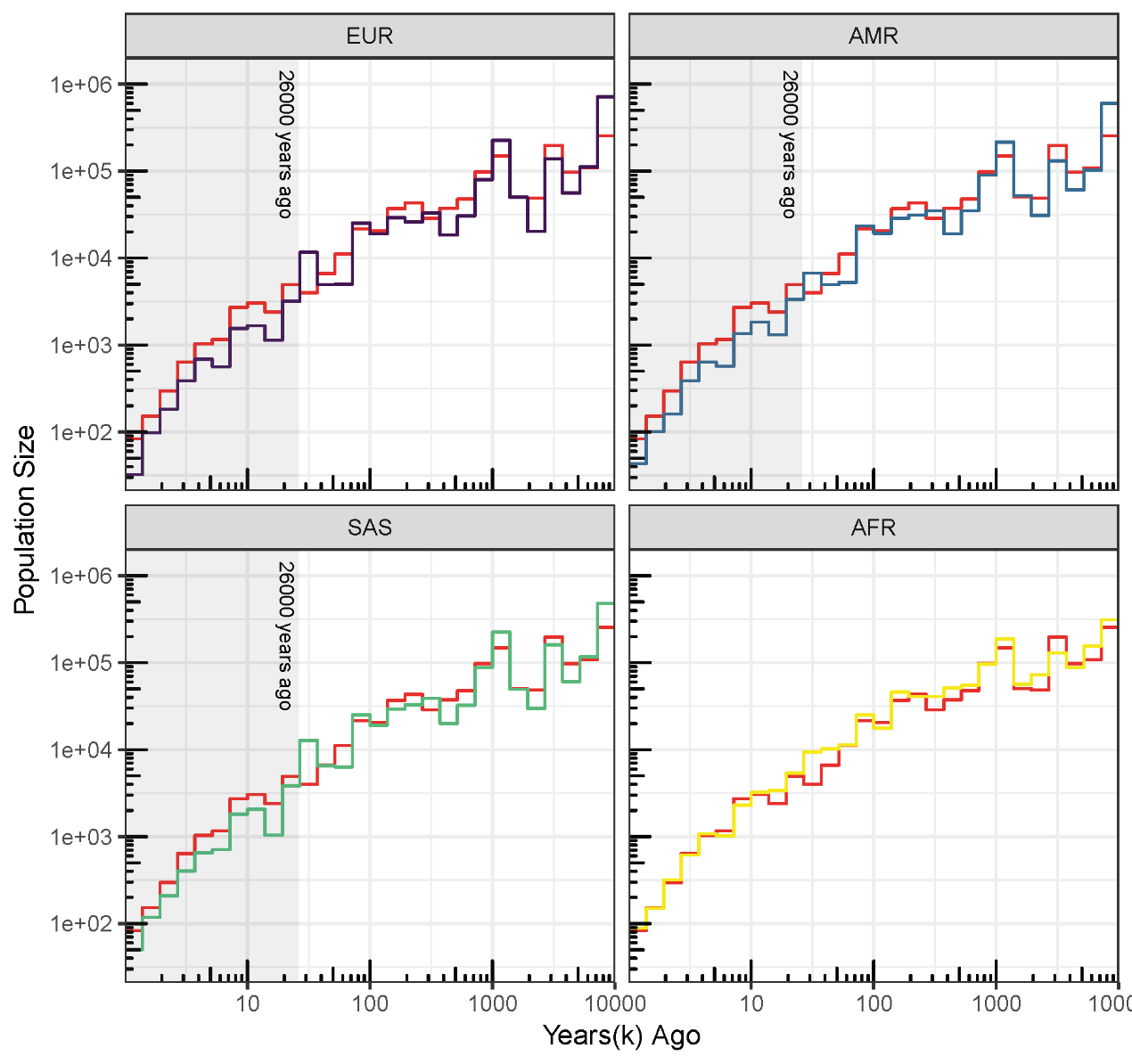


Figure S3: Effective population sizes inferred using Related-package across all individuals of each population in two loci (*HLA-DPA1, HLA-DPB1*). Recentsize histories (26000 years ago) in European (purple) population showed modest difference compared with East Asian (red) population. Abbreviation: EUR, European; AMR, American; SAS, South Asian; AFR, African.


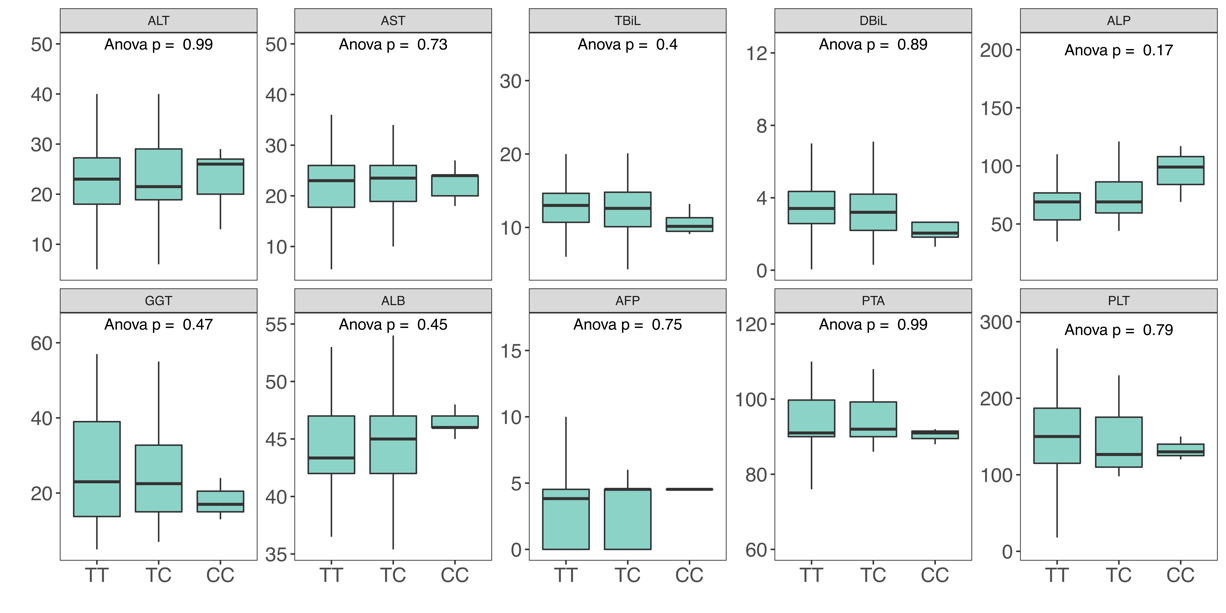


Figure S4: Boxplots of rs2395166 genotype and serum liver enzyme levels in HC.


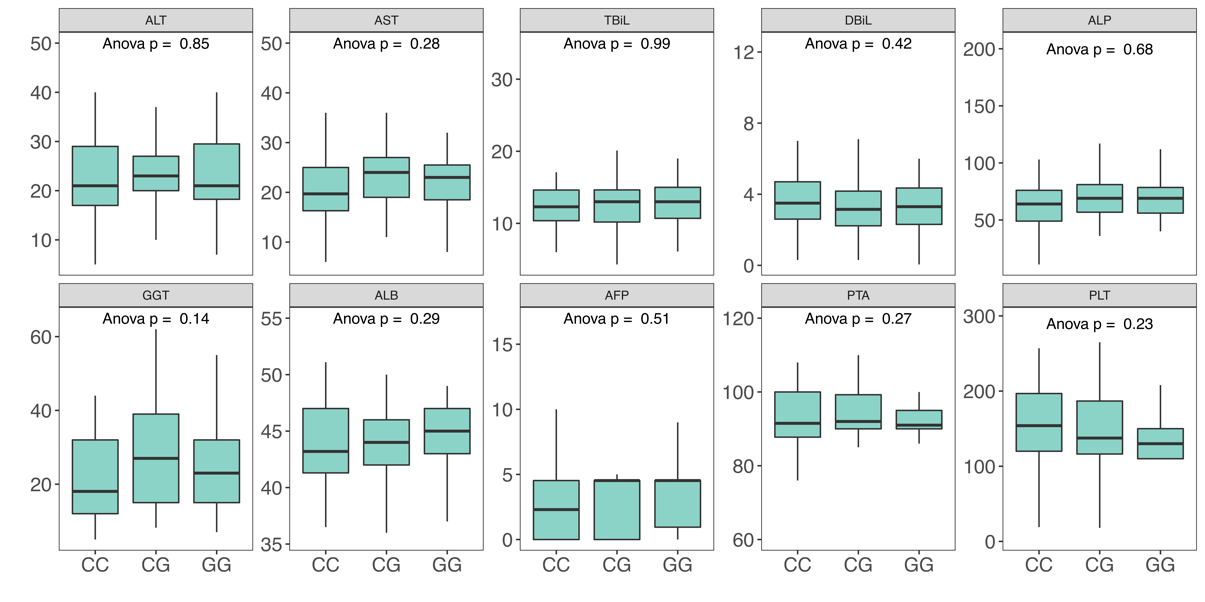


Figure S5: Boxplots of rs615672 genotype and serum liver enzyme levels in HC.


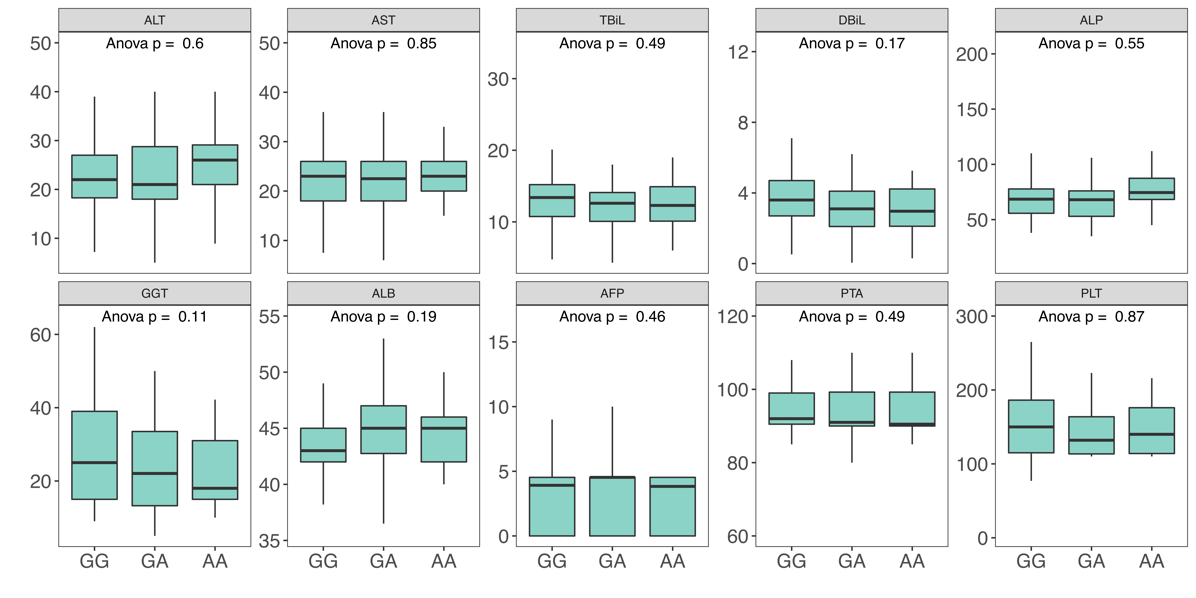


Figure S6: Boxplots of rs3077 genotype and serum liver enzyme levels in HC.


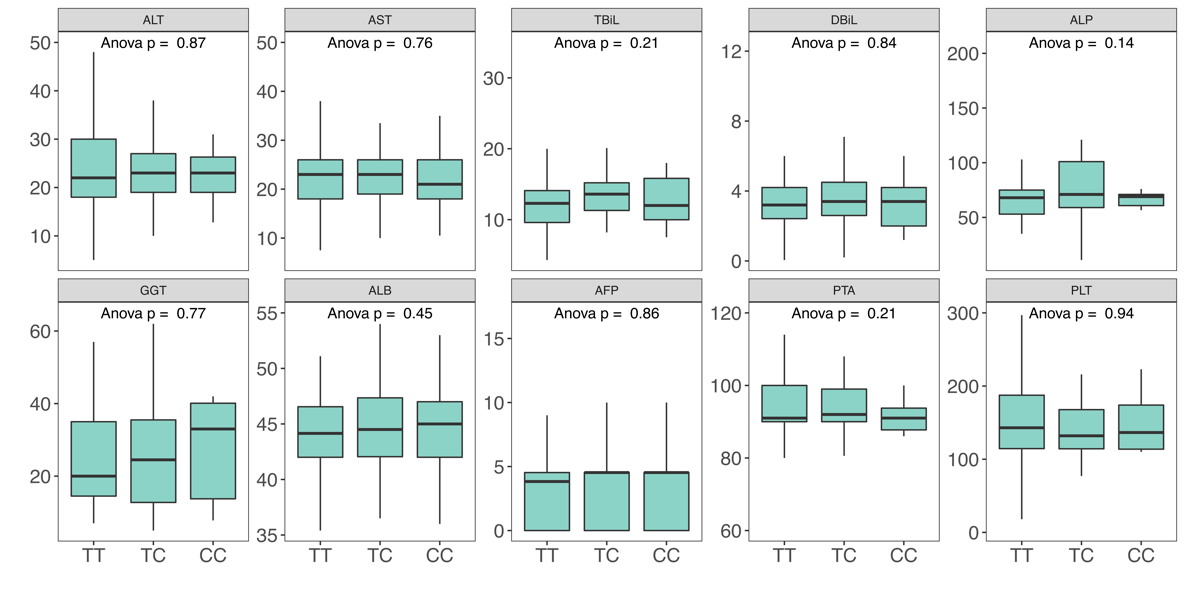


Figure S7: Boxplots of rs1264473 genotype and serum liver enzyme levels in HC.


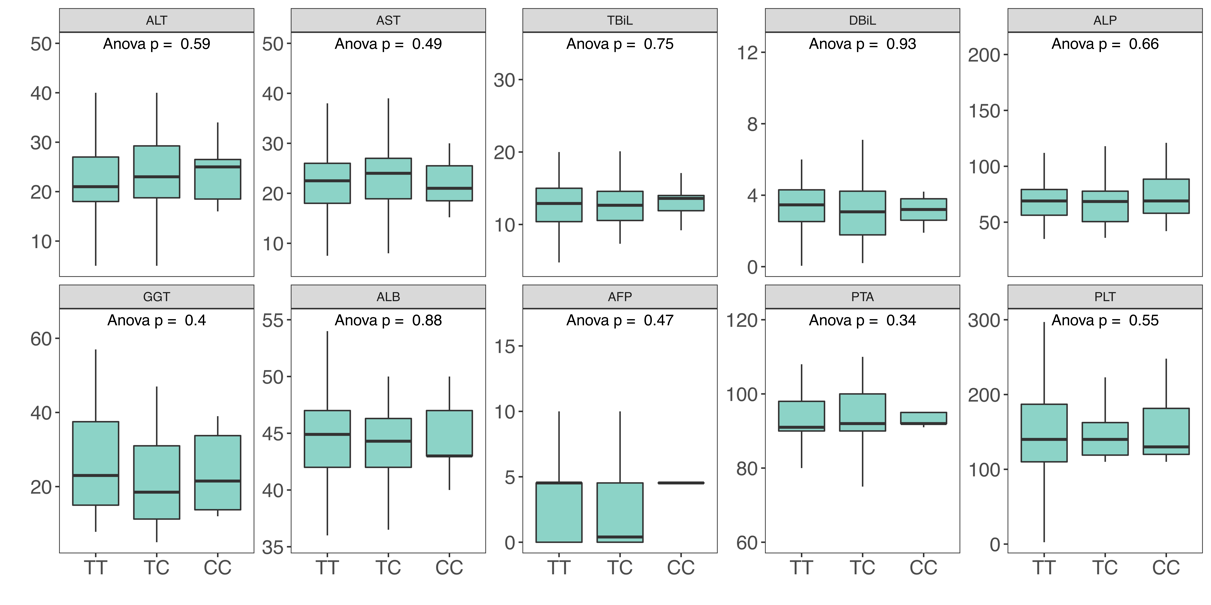


Figure S8: Boxplots of rs2833856 genotype and serum liver enzyme levels in HC.


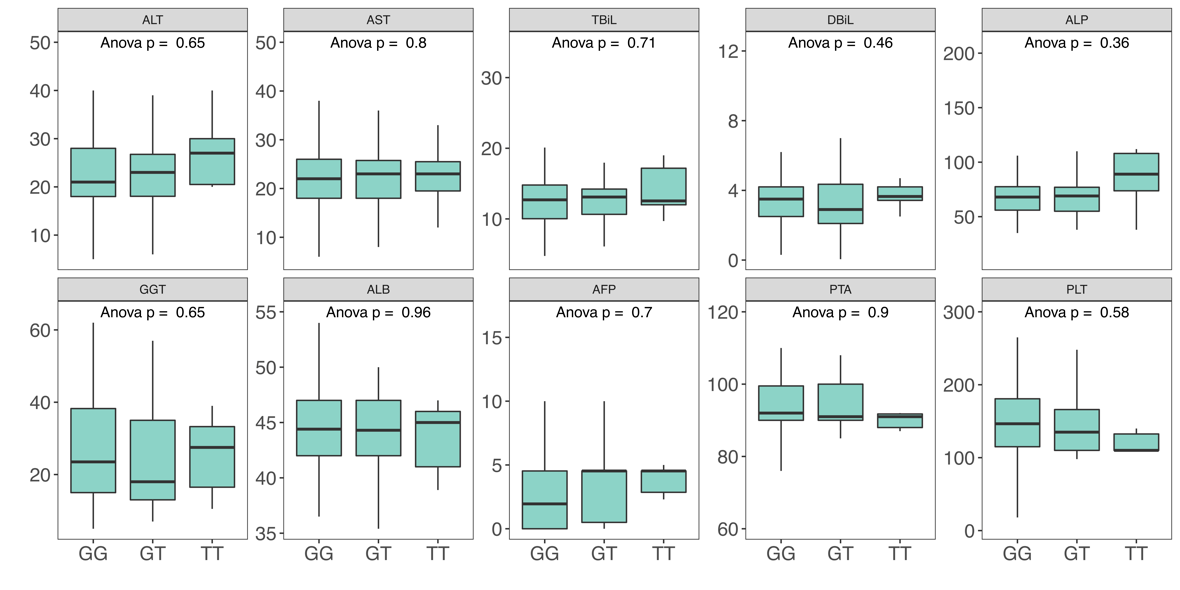


Figure S9: Boxplots of rs6942409 genotype and serum liver enzyme levels in HC.


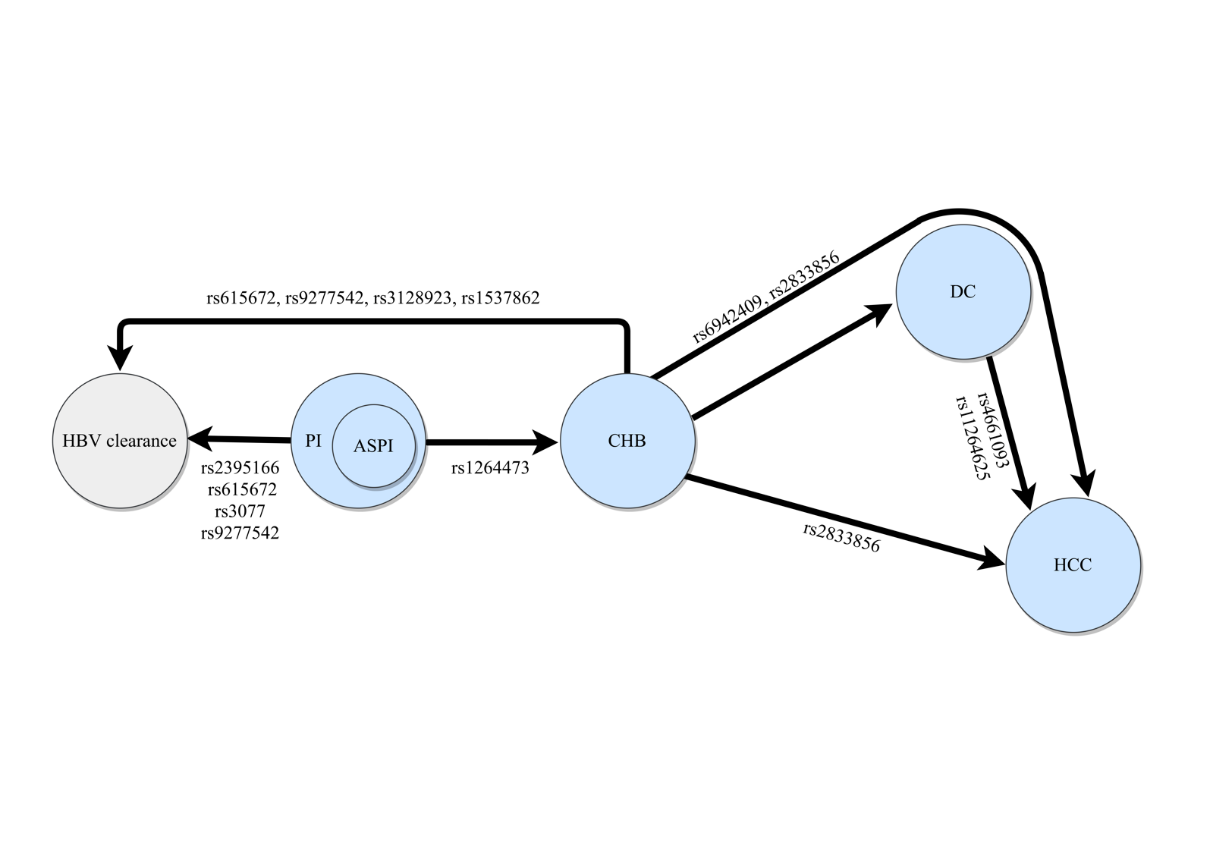


Figure S10: The summary of associated SNPs contributed to HBV-related outcomes and the progression. Abbreviation: PI, persistence infection; ASPI, asymptomatic persistence infection; CHB, chronic hepatitis B; DC, decompensated cirrhosis; HCC, hepatocellular carcinoma.
